# Detailed Chemical Prospecting of Volatile Organic Compounds Variations from Adriatic Macroalga *Halopteris scoparia*

**DOI:** 10.3390/molecules27154997

**Published:** 2022-08-05

**Authors:** Martina Čagalj, Sanja Radman, Vida Šimat, Igor Jerković

**Affiliations:** 1 Department of Marine Studies, University of Split, Ruđera Boškovića 37, 21000 Split, Croatia; 2 Department of Organic Chemistry, Faculty of Chemistry and Technology, University of Split, Ruđera Boškovića 35, 21000 Split, Croatia

**Keywords:** brown macroalgae, volatiles, gas chromatography and mass spectrometry (GC–MS), air-drying, benzaldehyde, pentadecane, pentadec-1-ene, (*E*)-phytol, diterpene alcohols

## Abstract

The present study aimed to isolate volatile organic compounds (VOCs) from fresh (FrHSc) and air-dried (DrHSc) *Halopteris scoparia* (from the Adriatic Sea) by headspace solid-phase microextraction (HS-SPME) and hydrodistillation (HD) and to analyse them by gas chromatography and mass spectrometry (GC–MS). The impact of the season of growth (May–September) and air-drying on VOC composition was studied for the first time, and the obtained data were elaborated by principal component analysis (PCA). The most abundant headspace compounds were benzaldehyde, pentadecane (a chemical marker of brown macroalgae), and pentadec-1-ene. Benzaldehyde abundance decreased after air-drying while an increment of benzyl alcohol after drying was noticed. The percentage of pentadecane and heptadecane increased after drying, while pentadec-1-ene abundance decreased. Octan-1-ol decreased from May to September. In HD-FrHSc, terpenes were the most abundant in June, July, and August, while, in May and September, unsaturated aliphatic compounds were dominant. In HD-DrHSc terpenes, unsaturated and saturated aliphatic compounds dominated. (*E*)-Phytol was the most abundant compound in HD-FrHSc through all months except September. Its abundance increased from May to August. Two more diterpene alcohols (isopachydictyol A and cembra-4,7,11,15-tetraen-3-ol) and sesquiterpene alcohol gleenol were also detected in high abundance. Among aliphatic compounds, the dominant was pentadec-1-ene with its peak in September, while pentadecane was present with lower abundance. PCA (based on the dominant compound analyses) showed distinct separation of the fresh and dried samples. No correlation was found between compound abundance and temperature change. The results indicate great seasonal variability of isolated VOCs, as well among fresh and dried samples, which is important for further chemical biodiversity studies.

## 1. Introduction

*Halopteris* is a genus of brown seaweeds that currently consists of 14 species. Species belonging to this order usually grow from 3 to 20 cm. Generally, they inhabit the lower intertidal zone and sublittoral in temperate regions. They grow on the rocks and have olive to dark-brown or reddish-brown thalli [1]. The extracts prepared from seaweeds belonging to *Halopteris* genus have shown various biological activities, including antiprotozoal activity [2,3], antibacterial activity [4,5,6,7,8], antifungal activity [7,8], apoptotic/cytotoxic activity [9], antioxidant activity [9,10,11,12], anti-inflammatory activity [12], anticoagulant activity [13], anti-acetylcholinesterase activity [14], and antifouling activity [7].

Nunes et al. [15] identified minor and major constituents in *Halopteris scoparia* harvested off the northern coast of the Island of Gran Canaria, Spain. The authors found 5.20 g/100 g dw (dry weight) of moisture, 57.20 g/100 g dw of total minerals, 5.54 g/100 g dw of proteins, 3.64 g/100 g dw lipids, and 29.86 g/100 g dw of total carbohydrates. As for the chlorophylls, carotenoids, and phenolic compounds, 8.15 mg/g dw, 2.15 mg/g dw, and 1.90 mg gallic acid equivalents (GAE)/g dw, respectively, were found. On the other hand, Uslu et al. [16] found 9.79 g/100 g dw of proteins, 2.85 g/100 g dw of lipids, and 28 g/100 g dw of ash from *H. scoparia* harvested from Iskenderun Bay, Turkey. Furthermore, the authors identified five fatty acids, myristic, palmitic, heptadecanoic, palmitoleic, and oleic acid, with palmitic acid as the predominant one with 37.47% of the total fatty acid content (TFAC) in *H. scoparia*. Pereira et al. [17] identified the fatty acid profile of *H. scoparia* harvested off the Algarve coast, Portugal. Palmitic acid was the predominant saturated fatty acid with 24.36% of TFCA. A high amount (51.01% of TFCA) of polyunsaturated fatty acids (PUFAs) was found. Linoleic, arachidonic, and eicosapentaenoic acids were the predominant PUFAs found with 20.35%, 13.96%, and 14.39% of TFCA, respectively. Docosahexaenoic acid was also identified. A similar fatty acid profile was found by Campos et al. [12] in *H. scoparia* harvested from Azores island, Portugal. Moreover, the authors prepared water extract from seaweed and found 217 mg GAE/100 g dw of phenolic compounds. Güner et al. [9] determined phenolic (33.20 mg GAE/g) and flavonoid (1.26 mg quercetin equivalent (QE)/g) contents in *H. scoparia* methanol extract. This seaweed was harvested off the coast of Urla, Turkey.

To the best of our knowledge, there are no records of *H. scoparia* volatile organic compound (VOC) composition. In our previous study, we identified VOCs from *Halopteris filicina* from the Adriatic Sea [18]. The most dominant individual compounds found were dimethyl sulphide, fucoserratene, benzaldehyde, and octan-1-ol with 12.8%, 9.5%, 8.7%, and 5.1%, respectively. Aliphatic compounds were the most dominant group. Furthermore, Whitfield et al. [19] identified bromophenols in two *Halopteris* species, *Halopteris paniculata* and *Halopteris platycena*, harvested from eastern Australia. Identified compounds were 2- and 4-bromophenol, 2,4- and 2,6-dibromophenol, and 2,4,6-tribromophenol. The methods used for *Halopteris* species VOC isolation were headspace solid-phase microextraction (HS-SPME) [18] and combined steam distillation solvent extraction (SDE) with pentane/diethyl ether (9:1 *v*/*v*) as the solvent after the alga was blended in purified water and acidified to pH 1 for the isolation of bromophenols [19]. Other methods were also used for algal VOC isolation in general such as hydrodistillation (HD) [20], purge and trap [21], simultaneous distillation extraction under reduced pressure, or accelerated solvent extraction [22].

This study aimed to isolate and analyse VOCs from fresh (FrHSc) and air-dried (DrHSc) *H. scoparia* harvested from the Adriatic Sea, by using HD and HS-SPME. These methods were selected since they are complementary, providing isolation of the headspace, volatile, and semi-volatile compounds. The use of HD is appropriate for volatile and semi-volatile compounds (discriminating the most volatile headspace compounds), and HS-SPME is a headspace method (discriminating semi-volatile compounds). Furthermore, the influence of air-drying and season of growth (May–September) on VOC composition was also studied for the first time, and the obtained data were elaborated by principal component analysis (PCA). It was expected to find pentadecane, a marker of brown macroalgae, along with other VOCs from different chemical groups, as well as to observe VOC seasonal changes and determine the impact of air-drying on VOC composition. Seasonal variability of algal VOCs is rarely studied in general, and we previously researched the seasonal variability of volatilome from *Dictyota dichotoma* [20].

## 2. Results and Discussion

### 2.1. Headspace Variations of H. Scoparia

**Figure 1:**
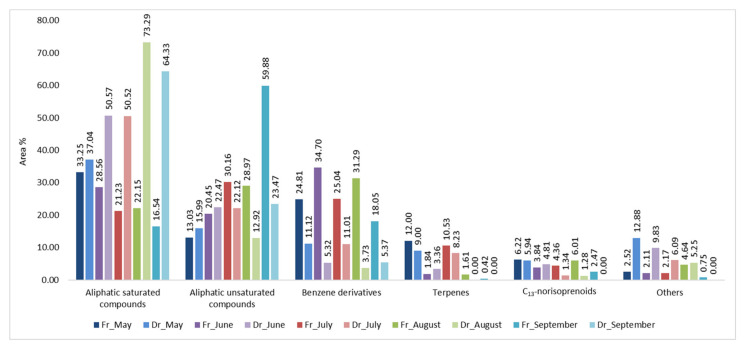
The VOCs from *H. scoparia* sorted by structural groups extracted by headspace solid-phase microextraction (HS-SPME) and analysed by gas chromatography–mass spectrometry (GC–MS) using DVB/CAR/PDMS fibre (f1); Fr (fresh), Dr (dry).

**Figure 2:**
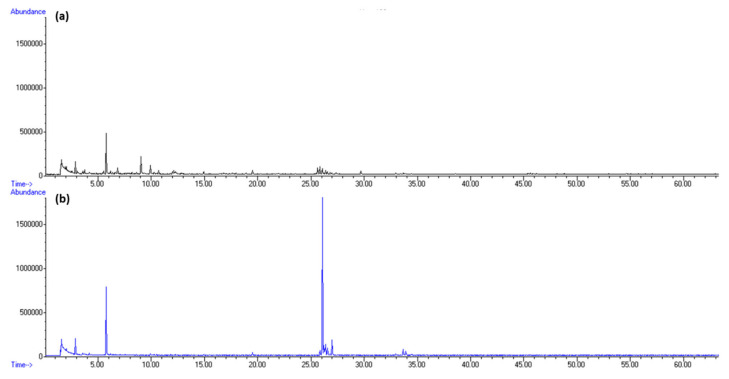
Total ion chromatogram (TIC) comparison of *H. scoparia* isolated by headspace solid-phase microextraction (HS-SPME) and analysed by gas chromatography–mass spectrometry (GC–MS) using DVB/CAR/PDMS fibre (f1): (**a**) in May; (**b**) in September.

**Figure 3:**
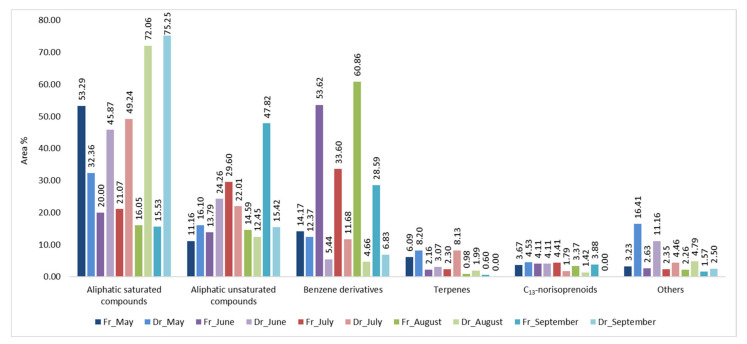
The VOCs from *H. scoparia* sorted by structural groups extracted by headspace solid-phase microextraction (HS-SPME) and analysed by gas chromatography–mass spectrometry (GC–MS) using PDMS/DVB fibre (f2); Fr (fresh), Dr (dry).

**Figure 4:**
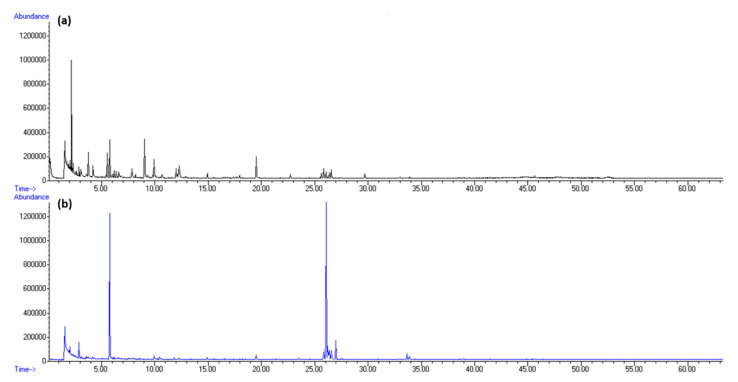
Total ion chromatogram (TIC) comparison of *H. scoparia* isolated by headspace solid-phase microextraction (HS-SPME) and analysed by gas chromatography–mass spectrometry (GC–MS) using PDMS/DVB fibre (f2): (**a**) in May; (**b**) in September.

Headspace composition was analysed using HS-SPME. To acquire more information about the headspace composition two fibres of different polarities were used: divinylbenzene/carboxene/polydimethylsiloxane (DVB/CAR/PDMS, f1) and polydimethylsiloxane/divinylbenzene (PDMS/DVB, f2). According to our previous research [20,23,24], HS-SPME provides a large number of extracted headspace compounds with more or less diversity within two fibres of different polarity. In FrHSc headspace (HS-FrHSc), great variability was found within the months by two fibres, while the DrHSc headspace (HS-DrHSc) volatilome was more comparable. From 91.50% (HS-FrHSc, June) to 99.31% (HS-DrHSc, July) of VOCs were identified with f1 and from 89.97% (HS-DrHSc, May) to 100% (HS-DrHSc, September) of VOCs were identified with f2. Identified VOCs could be classified into six different groups: saturated aliphatic compounds, unsaturated aliphatic compounds, benzene derivatives, terpenes, C_13_-norisoprenoids (carotenoids degradation products), and others (Figure 1 and Figure 2). The dominant VOCs were aliphatic compounds followed by benzene derivatives, except for HS-FrHSc in June and August extracted with f2, where benzene derivatives represented the most prevalent group of compounds (Figure 3 and Figure 4).

The most abundant headspace compounds were benzaldehyde (HS-FrHSc in May (23.85%, f1), June (34.24%, f1; 52.75%, f2), July (32.99%, f2), and August (31.29%, f1; 57.85%, f2), pentadecane (HS-DrHSc from May (22.06%, f1; 14.06%, f2) to September (29.33%, f1; 24.47%, f2)), and pentadec-1-ene (HS-FrHSc in July (25.14%, f1) and September (53.17%, f1; 40.35%, f2)). Benzaldehyde abundance decreased after air-drying. Analysing with f1, the higher drop was in June (8.8 times) and August (8.4 times), while, analysing with f2, this decrease was even more perceived—June (13.8 times) and August (22.1 times). Since benzaldehyde is highly volatile, its loss after air-drying could be assigned to this property [24]. In the period from May to July, an increment in benzyl alcohol area percentage after the drying was noticed, with a peak in May (5.84%, f1; 6.53%, f2). Analysing with f1, it was not detected in HS-FrHSc within all months and also not in HS-DrHSc in August and September (as also seen for f2) (Table 1). This could be the result of polyphenolic compound oxidation rather than lignin-like compound degradation since brown algae seem to not specifically contain lignin, but only phenol compounds [25,26].

The area percentage of two saturated aliphatic hydrocarbons, pentadecane and heptadecane, increased after air-drying in all months (analysed with both fibres; Table 1 and Table 2). The largest increment in pentadecane was noticed in May (6.2 times, f1; 7.2 times, f2) and September (7.2 times, f1; 9.1 times, f2), while, for heptadecane, it was in August (4.2 times, f1; 21.8 times, f2) and September (8.9 times, f1; 22.7 times, f2). This could be the consequence of fatty acid degradation [24]. Pentadec-1-ene, as the most abundant unsaturated hydrocarbon, decreased during all months after air-drying, with the top variation in July (13.0 times, f1;12.4 times, f2). Octan-1-ol showed the greatest change through the months. It was detected only in HS-FrHSc with the greatest abundance in May (13.35%, f1; 13.93%, f2). In June, its portion dropped by 4.7 times on f1 and 4.6 times on f2 and even more in July (2.4 times more on f1; 3.8 times more on f2). Similar behaviour was noticed for unsaturated ketones 3-hydroxybutan-2-one (detected with f2) and 6-methylheptan-2-one.

Among terpenes, sesquiterpene germacrene D and diterpene phytane were dominant. Germacrene D was detected only in HS-FrHSc with the peak in May (5.44%, f1; 1.87%, f2). In our previous research, germacrene D was found in *D. dichotoma* headspace as one of the most abundant terpenes at the blooming inception in May [20]. Phytane was detected only in July with the greatest portion in HS-DrHSc (4.46%, f1; 4,19%, f2). The increment in its abundance after drying implies possible chlorophyll degradation. Total ion chromatograms (TICs) of fresh samples from May and September isolated by HS-SPME are presented in Figure 2 for fibre f1 and Figure 4 for fibre f2.

### 2.2. Statistical Analysis of the H. scoparia Headspace VOCs

**Figure 5:**
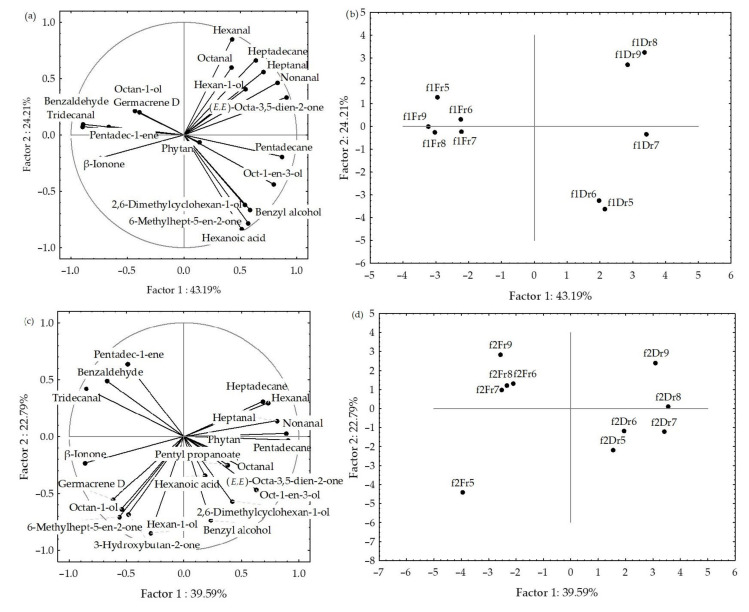
Correlation loadings (**a**,**c**) and score plots (**b**,**d**) of the dominant compounds from the headspace volatiles obtained by two different fibres for HS-SPME/GC–MS: divinylbenzene/carboxene/polydimethylsiloxane (f1) and polydimethylsiloxane/divinylbenzene (f2) of fresh (Fr, **a**,**b**) and dried (Dr, **c**,**d**) *H. scoparia* samples.

**Figure 6:**
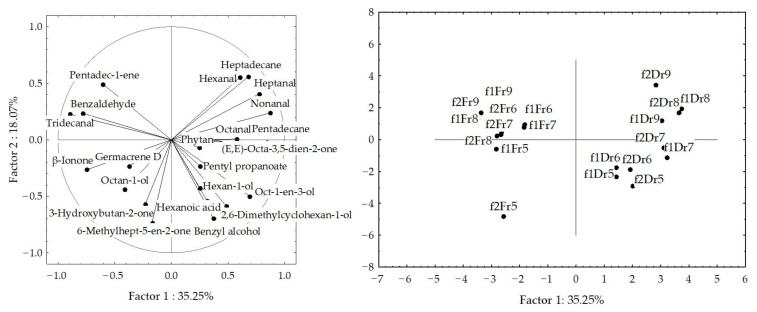
Correlation and score plots of the dominant compounds from headspace volatiles (two different fibres for HS-SPME/GC–MS: divinylbenzene/carboxene/polydimethylsiloxane (f1) and polydimethylsiloxane/divinylbenzene (f2)) of fresh (Fr) and dried (Dr) *H. scoparia* samples.

The principal component analyses (PCA) were used to describe the variations among the dominant volatiles (>2%) of HS-FrHSc and HS-DrHSc in relation to the material preparation (fresh or dry), seasonal changes, and fibre. The results are shown in Figure 5 and Figure 6.

The PCA analysis of the data obtained by f1 fibre is shown in Figure 2a,b. The first two PCs described 67.4% of the initial data variability. A correlation between certain groups of the compounds was observed (Figure 5a). Pentadecane, nonanal, β-ionone, and tridecanal showed the highest variable contribution to PC1 and factor-variable correlation, while hexan-1-ol and benzyl alcohol contributed to PC2.

The correlation plot and score plot of the dominant components from data obtained by f2 fibre are shown in Figure 5c,d. The first two PCs described 62.38% of the initial data variability. The abundance of benzaldehyde, (*E,E*)-octa-3,5-dien-2-one, pentadecane, and tridecanal contributed to PC1, while hexanal and hexanoic acid had the highest contribution to PC2. May samples (fibre f2) were separated in the bottom left part of the plot as a function of the highest octan-1-ol and 3-hydroxybutan-2-one content.

A clear separation between the fresh and dried samples was obtained for both fibres (Figure 5b,d). For f1, fresh samples were positioned on the left part of the multivariate space, while the scores of the dried samples were vertically distributed on the right side of the score plot. Interestingly, the sampling month showed no effect on the fresh samples, while, in dry samples, there were similarities between May and June, and between August and September. Similarly, a clear separation of the dry and fresh samples was observed for f2 fibre. As it can be seen in Figure 5d, the fresh sample harvested in May showed great variation compared to other months. The main reason was the high portion of aliphatic saturated compounds (53.29%), as well as terpenes (6.09%) and others (3.23%).

When the data for f1 and f2 were analysed together, the first two PCs described 53.3% of the initial data variability, but the score plot showed a clear separation between fresh and dried samples (Figure 6). As a function of the higher content of benzaldehyde and octan-1-ol, as well as low content of pentadec-1-ene and pentadecane in the dry samples, the samples from May (both fibres) were segregated in the bottom part of the plot and September samples in the top part. Some separation between the sampling months was seen, but no correlation with temperature change over months was observed.

### 2.3. Volatile Organic Compounds Variations of H. scoparia Acquired by Hydrodistillation

**Figure 7:**
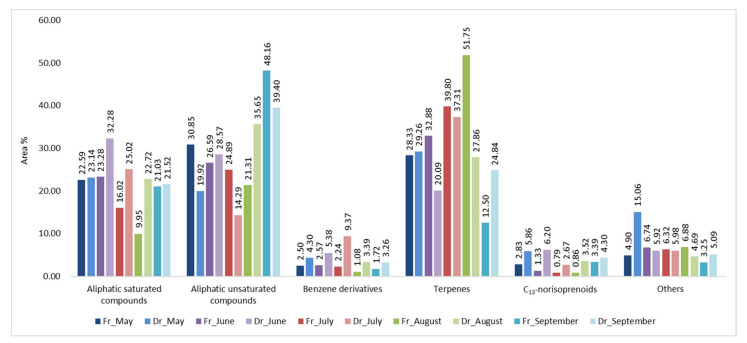
The VOCs from *H. scoparia* sorted by structural groups obtained by hydrodistillation (HD) and analysed by gas chromatography–mass spectrometry (GC–MS); Fr (fresh), Dr (dry).

In the hydrodistillate (HD), the portion of the identified compounds ranged from 90.04% (HD-FrSc, September) to 98.44% (HD-DrHSc, June) of the total detected compounds. Identified VOCs could be classified, as for the headspace, into six different groups: saturated aliphatic compounds, unsaturated aliphatic compounds, benzene derivatives, terpenes, C_13_-norisoprenoids, and others (Figure 7). In HD-FrHSc, terpenes were the most abundant group in June (32.88%), July (39.80%), and August (51.75%), while, in May (30.85%) and September (48.16%), the dominant group was unsaturated aliphatic compounds. In HD-DrHSc, three groups of compounds dominated through the months: terpenes (May, 29.26%; July, 37.31%), unsaturated aliphatic compounds (August, 35.65%; September, 39.40%), and saturated aliphatic compounds (June, 32.28%).

Diterpene alcohol (*E*)-phytol was the most abundant compound in HD-FrHSc through all months except September. Its abundance increased from May (11.35%) to August (47.03%) when it was in its highest bloom. Recent studies have shown its antioxidant potential [27], antimicrobial [28], anti-inflammatory [29], antitumour [30], and antidiabetic activities [27]. Its derivative, phytane, was detected in July (6.11%, HD-FrHSc; 2.43%, HD-DrHSc). Two more diterpene alcohols, isopachydictyol A and cembra-4,7,11,15-tetraen-3-ol, and one sesquiterpene alcohol, gleenol, were detected in high abundance. Both diterpene alcohols were the most abundant in May (9.70%, isopachydictyol A; 6.88%, cembra-4,7,11,15-tetraen-3-ol) in the sample after air-drying and were detected in the lowest content in the fresh sample in September. They were detected as the major compounds in HD of *D. dichotoma* [20] and isolated from several species of *Dictyota* genus [31,32]. Both diterpenes have various biological activity [33,34]. Gleenol was detected in the highest portion in May in HD-FrHSc (6.26%) and its portion decreased each month (Table 3).

**Figure 8:**
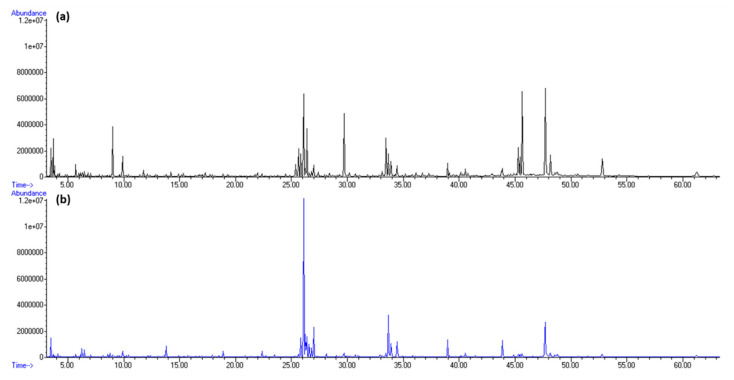
Total ion chromatogram (TIC) comparison of *H. scoparia* obtained by hydrodistillation (HD) and analysed by gas chromatography–mass spectrometry (GC–MS): (**a**) in May; (**b**) in September.

Among aliphatic compounds, the dominant was unsaturated pentadec-1-ene with its peak in September in both HD-FrHSc (26.46%) and HD-DrHSc (18.98%). Higher aliphatic unsaturated alcohol (*Z,Z,Z*)-octadeca-9,12,15-trien-1-ol was prevalent in HD-FrHSc in May (8.09%) and decreased each month. Higher aliphatic unsaturated aldehyde (*Z*)-octadec-9-enal was the most abundant in HD-FrHSc in August (8.51%). Both the alcohol and the aldehyde were less abundant in air-dried samples throughout all the months since lipid oxidative degradation probably occurred, which means they were oxidised and further degraded. Six carboxylic acids were detected and identified with hexanoic acid as the most abundant one particularly in HD-DrHSc in May (4.80%). Three C_13_-norisoprenoids were detected, among which β-ionone was the most abundant. It was higher in air-dried samples, which was the result of carotenoid degradation during the process of air-drying. Total ion chromatogram (TIC) of fresh samples from May and September obtained by HD are presented in Figure 8.

### 2.4. Statistical Analysis of the H. scoparia VOCs Obtained by Hydrodistillation

**Figure 9:**
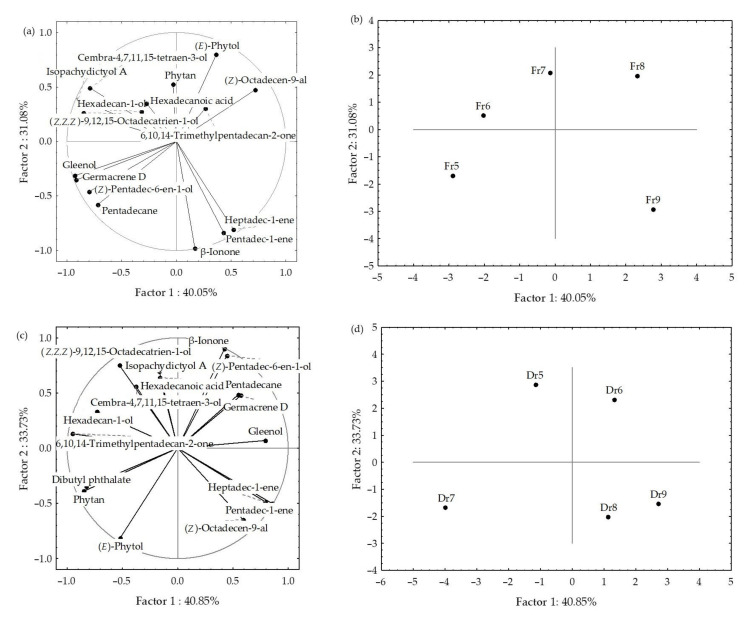
Correlation loadings (**a**,**c**) and score plot (**b**,**d**) of the dominant VOCs of fresh (Fr, **a**,**b**) and air-dried (Dr, **c**,**d**) *H. scoparia* samples obtained by hydrodistillation.

**Figure 10:**
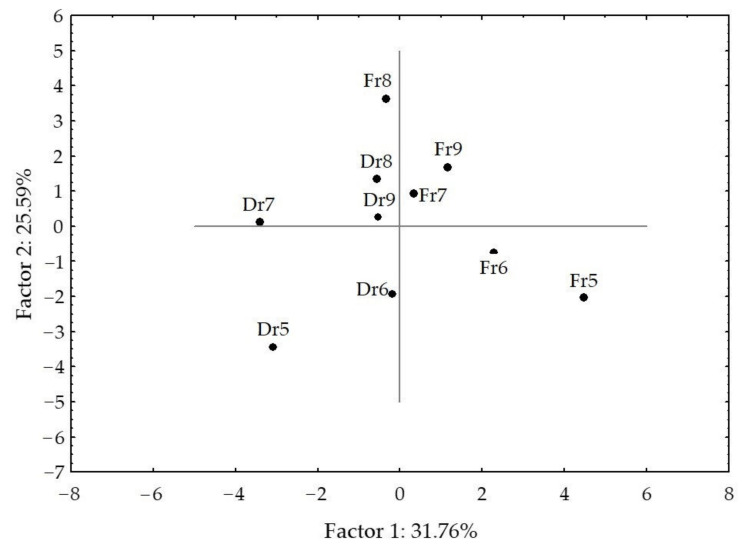
Score plot of the dominant VOCs of fresh (Fr) and air-dried (Dr) *H. scoparia* samples obtained by hydrodistillation.

The PCA results for VOCs of fresh and air-dried *H. scoparia* obtained by hydrodistillation are shown in Figure 9 and Figure 10.

In fresh samples, the first two PCs described 71.13% of the initial data variability. The correlation loadings of the first two PCs (Figure 9a) showed high correlations between germacrene D and (*Z*)-pentadec-6-en-1-ol and gleenol, between heptadec-1-ene and pentadec-1-ene, and between hexadecan-1-ol and hexadecanoic acid. Germacrene D, gleenol, (*Z,Z,Z*)-octadeca-9,12,15-trien-1-ol, and isopachydictyol A were the variables with the highest variable contributions, according to the correlations. The PC2 was associated with β-ionone, pentadec-1-ene, heptadec-1-ene, and (*E*)-phytol abundance in the samples. The score plot (Figure 9b) showed the position samples in the multivariate space of the first two PCs. There was a clear separation between the months. The dominant group in the fresh samples from May and September was aliphatic unsaturated compounds, while, in June, July, and August, it was the group of terpenes.

In dried samples, aliphatic compounds (pentadec-1-ene, phytane, and 6,10,14-trimethylpentadecan-2-one) were variables with the highest contribution to PC1, while β-ionone, (*Z*)-pentadec-6-en-1-ol, and (*E*)-phytol, contributed the most to PC2. High correlations were observed among germacrene D, pentadecane, and (*Z*)-pentadec-6-en-1-ol, as well as between phytane and dibutyl phthalate.

When all dominant VOCs obtained by hydrodistillation were analysed, a clear separation of fresh and dried samples was observed (Figure 10). May and June samples were positioned in the lower part of the plot, but clearly separated on the basis of the difference in the content of β-ionone, gleenol, (*Z,Z,Z*)-octadeca-9,12,15-trien-1-ol, and (*E*)-phytol. There were more similarities in seasonal variation between dry samples. The dried samples appeared in the left part of the plot. The distribution was along the PC1 axis and was in the relation to aliphatic compounds (germacrene D, pentadecane, gleenol, and 6,10,14-trimethylpentadecan-2-one), while the distribution along the PC2 axis was related to sesquiterpene alcohols (cembra-4,7,11,15-tetraen-3-ol and gleenol) and methyl (*Z*)-pentadec-6-en-1-ol, (*Z*)-octadec-9-enal, (*Z,Z,Z*)-octadeca-9,12,15-trien-1-ol, (*E*)-phytol, isopachydictyol A, and cembra-4,7,11,15-tetraen-3-ol abundance in the samples. No correlation was found between the compound abundance and temperature change.

The impact of HS-SPME and HD on the obtained results was obvious from the composition of the headspace (Table 1 and Table 2) and hydrodistillate (Table 3) of the fresh samples. The most abundant headspace compounds were benzaldehyde, pentadecane, and pentadec-1-ene. (*E*)-Phytol was the most abundant compound in HD-FrHSc (except September) followed by diterpene alcohols (isopachydictyol A and cembra-4,7,11,15-tetraen-3-ol) and sesquiterpene alcohol gleenol.

The used statistical test did not correlate compound abundance with the temperature change as the temperature varied.

## 3. Materials and Methods

### 3.1. Sample Collection

**Figure 11:**
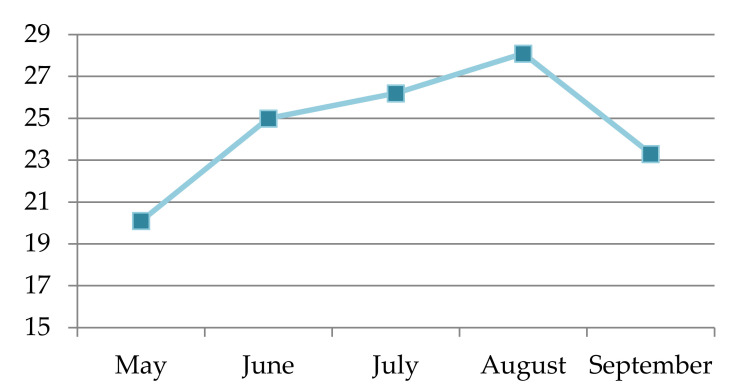
Sea temperature measured during harvesting.

The seaweed *H. scoparia* (Linnaeus) Sauvageau 1904 samples were harvested in 2021 from May to September. The sampling location was in the Adriatic Sea on the southern side of the island Čiovo (43.493373° N, 16.272519° E). Seaweed samples were harvested at a depth ranging from 20 to 120 cm. A YSI Pro2030 probe (Yellow Springs, OH, USA) was used to measure the sea temperature (Figure 11). The identification was performed by a marine botanist according to seaweed morphological characteristics. The samples were air-dried in the shade at room temperature for 10 days following a similar pattern, which is very common for terrestrial plants and similar to our other studies [20,23,24].

### 3.2. Headspace Solid-Phase Microextraction (HS-SPME) 

Two SPME fibres (Agilent Technologies, Palo Alto, Santa Clara, CA, USA) covered with PDMS/DVB (polydimethylsiloxane/divinylbenzene) or DVB/CAR/PDMS (divinylbenzene/carboxene/polydimethylsiloxane) and placed on the PAL Auto Sampler System (PAL RSI 85, CTC Analytics AG, Schlieren, Switzerland) were used for HS-SPME. Prior to the extraction, both fibres were conditioned following the manufacturer instructions. The seaweed samples (1 g) were placed into 20 mL glass vials sealed with a stainless-steel cap with polytetrafluorethylene (PTFE)/silicon septa. The equilibration of the sample was conducted at 60 °C for 15 min. After equilibration, the sample was extracted for 45 min. Thermal desorption directly to the GC column was carried out at an injector temperature of 250 °C set for 6 min.

### 3.3. Hydrodistillation (HD)

Approximately 50 g of fresh and 20 g of air-dried *H. scoparia* samples were hydrodistilled. For HD, a *v/v* ratio 1:2 (3 mL) of pentane and diethyl ether was used as the solvent trap in a modified Clevenger apparatus. HD was performed for 2 h, and the solvent trap with dissolved VOCs was isolated. Concentration of the solvent trap was performed under a slow flow of nitrogen until the final volume was 0.2 mL. A volume of 2 µL was used for GC–MS analyses.

### 3.4. Gas Chromatography–Mass Spectrometry Analysis (GC–MS) 

VOCs isolated from *H. scoparia* were analysed using a gas chromatograph (model 8890 Agilent Technologies, Palo Alto, Santa Clara, CA, USA) equipped with a mass spectrometer detector (model 5977E MSD, Agilent Technologies). For the separation of VOCs, HP-5MS capillary column (30 m × 0.25 mm, 0.25 µm film thickness, Agilent Technologies, Palo Alto, Santa Clara, CA, USA) was used. The conditions for the GC–MS analysis and the process for the identification of the compounds were previously described in detail by Radman et al. [20]. The analyses were carried out in duplicate and shown as the average percentage of peak area.

### 3.5. Statistical Analyses

The relations between the dominant volatiles (>2%) of fresh and dried alga samples, analysed by HS-SPME and HD were determined using principal component analysis (PCA) [35]. The average percentage peak areas of the dominant volatiles from fresh and dried samples obtained by HS-SPME (two fibres) and HD were used for the analysis. For the PCA analyses, STATISTICA^®^ (version 13, StatSoft Inc, Tulsa, OK, USA) was used, and the data were log-transformed prior to analyses.

## 4. Conclusions

The present study reported complementary isolation of VOCs from FrHSc and DrHSc by headspace solid-phase microextraction (HS-SPME) and hydrodistillation (HD) and their analysis by gas chromatography and mass spectrometry (GC–MS). The use of both methods was justified for detailed chemical prospection of the headspace, volatile, and less volatile organic compounds from *H. scoparia*. The impact of the season of growth (May–September) and air-drying on VOC composition was also studied for the first time, and the obtained data were elaborated by principal component analysis (PCA). 

The most abundant headspace compounds of *H. scoparia* were benzaldehyde, pentadecane (a chemical marker of brown macroalgae), and pentadec-1-ene. Benzaldehyde abundance decreased after air-drying, while an increment in benzyl alcohol after the drying was noticed. The percentage of pentadecane and heptadecane increased after air-drying while pentadec-1-ene abundance decreased. Octan-1-ol decreased from May to September. In HD-FrHSc, terpenes were the most abundant group in June, July, and August, while, in May and September, the dominant group was unsaturated aliphatic compounds. In HS-FrHSc, great variability was found within the 5 months by two fibres, while the HS-DrHSc volatilome was more comparable.

In HD-DrHSc terpenes, unsaturated and saturated aliphatic compounds dominated. (*E*)-phytol was the most abundant compound in HD-FrHSc through all months except September. Its abundance increased from May to August. Since phytol is a bioactive compound, the obtained hydrodistillates could be attractive for further bioactivity studies. Two more diterpene alcohols, isopachydictyol A and cembra-4,7,11,15-tetraen-3-ol, and one sesquiterpene alcohol, gleenol, were also detected in high abundance. However, due to lower volatility, phytol and other diterpene alcohols could not be isolated completely by HD. Among aliphatic compounds, the dominant was pentadec-1-ene with its peak in September, while pentadecane was present with lower abundance. 

PCA (based on the dominant compound analyses) showed a distinct separation of the fresh and dried samples. However, the used statistical test did not correlate compound abundance with the temperature change as the temperature varied. The results indicate great seasonal variability of isolated VOCs, as well among fresh and dried samples, which is important for further chemical biodiversity studies.

## Figures and Tables

**Table 1:** The volatile compounds from *H. scoparia* isolated by headspace solid-phase microextraction (HS-SPME) and analysed by gas chromatography–mass spectrometry (GC–MS) using DVB/CAR/PDMS fibre (f1); Fr (fresh), Dr (dry).

Compound	RI	Area %									
		May		June		July		August		September	
		Fr	Dr	Fr	Dr	Fr	Dr	Fr	Dr	Fr	Dr
Dimethyl sulfide	<900	-	-	-	-	-	0.76	-	-	-	-
2-Methylpropan-1-ol	<900	-	0.57	-	-	-	-	-	-	-	-
3-Methylbutanal	<900	0.57	-	0.49	-	0.71	-	0.24	-	-	-
Pent-1-en-3-ol	<900	0.97	1.12	1.09	2.34	0.60	1.44	0.48	0.72	0.44	1.54
2-Methylbutanol	<900	-	1.04	-	-	-	-	-	-	-	-
Pentan-1-ol	<900	-	0.24	-	0.55	-	0.47	-	0.79	-	-
(*Z*)-Pent-2-en-1-ol	<900	0.48	-	0.34	0.52	-	0.61	-	-	-	-
Hexanal	<900	3.80	1.83	3.39	1.60	2.03	4.00	1.99	6.11	2.60	4.58
3-Methylbutanoic acid	<900	-	1.13	-	0.90	-	-	-	-	-	-
2-Methylbutanoic acid	<900	-	0.70	-	0.42	-	-	-	-	-	-
(*E*)-Hex-2-enal	<900	1.11	1.32	0.88	0.40	0.39	0.59	0.42	-	0.38	-
Hexan-1-ol	<900	1.26	0.87	0.44	0.95	0.94	3.15	1.29	1.19	-	5.61
Tribromomethane	<900	-	1.80	-	0.77	-	-	0.72	0.93	0.44	-
Heptan-2-one	<900	-	0.39	-	0.16	-	1.16	-	1.70	-	-
Heptanal	907	-	1.12	-	0.67	-	2.69	-	8.83	-	3.53
6-Methylheptan-2-one	962	1.93	-	1.39	0.39	0.23	0.32	-	-	-	-
Benzaldehyde	970	23.85	5.29	34.24	3.91	24.63	6.23	31.29	3.73	18.05	5.37
Heptan-1-ol	975	-	0.69	-	0.56	-	0.51	-	-	-	-
3,5,5-Trimethylhex-2-ene	980	-	-	0.71	-	0.73	-	-	-	-	-
Hexanoic acid	981	-	3.40	-	2.90	-	1.49	-	-	-	-
Sabinene	982	-	-	-	-	0.39	-	-	-	-	-
Oct-1-en-3-ol	984	1.44	3.79	1.00	6.77	0.78	5.36	0.55	2.54	-	2.92
Octan-3-one	991	1.13	-	1.50	-	0.50	-	-	-	-	-
6-Methylhept-5-en-2-one	992	-	1.49	-	1.10	-	1.09	-	-	-	-
Octan-2-one	995	-	-	0.52	-	0.57	-	1.04	-	-	-
2-Pentylfuran	996	-	1.05	-	0.91	-	2.17	-	1.89	-	-
Octanal	1007	3.10	0.99	1.40	-	0.66	2.92	-	3.88	-	1.59
(*E,E*)-Hepta-2,4-dienal	1017	-	-	-	0.39	-	1.41	-	-	-	-
2-Ethylhexan-1-ol	1035	-	-	-	0.47	-	2.09	-	1.14	-	-
(*E*)-3-Ethyl-2-methylhexa-1,3-diene	1038	-	1.41	-	1.49	-	1.38	-	-	-	-
Benzyl alcohol	1042	-	5.84	-	1.41	-	4.77	-	-	-	-
(*E*)-β-Ocimene	1044	-	2.64	-	1.31	1.27	1.95	-	-	-	-
Phenylacetaldehyde	1052	0.96	-	0.46	-	0.41	-	-	-	-	-
(*E*)-Oct-2-enal	1064	0.96	-	0.53	0.62	0.62	1.50	0.68	0.98	-	-
(*E*)-Oct-2-en-1-ol	1074	-	-	-	0.81	-	0.76	-	-	-	-
Octan-1-ol	1076	13.35	-	2.84	-	1.17	-	-	-	-	-
(*E,E*)-Octa-3,5-dien-2-one	1077	-	1.35	-	1.76	-	3.63	-	3.41	-	4.59
(*E,Z*)-Octa-3,5-dien-2-one	1097	-	2.29	-	2.25	-	2.43	-	0.74	-	-
Nonanal	1108	1.08	2.34	0.69	0.46	0.56	5.20	-	5.85	0.24	4.17
2,6-Dimethylcyclohexan-1-ol	1114	-	4.54	0.89	3.66	0.71	1.66	0.52	2.43	0.31	-
4-Ketoisophorone	1150	-	2.63	-	1.11	-	-	-	-	0.27	-
(*Z*)-Non-3-en-1-ol	1155	1.75	-	-	-	-	-	-	-	-	-
6-[(1*Z*)-Butenyl]-cyclohepta-1,4-diene] (Dictyopterene D’)	1158	2.52	0.27	1.21	0.27	1.46	-	2.49	-	-	-
[6-Butyl-cyclohepta-1,4-diene] (Dictyopterene C’)	1175	-	-	-	-	-	-	0.92	-	-	-
Decan-2-one	1196	-	-	-	-	-	-	0.65	-	-	-
Decanal	1209	-	-	-	-	-	0.92	-	1.13	-	-
β-Cyclocitral	1226	2.34	1.06	1.06	0.78	1.03	-	1.25	-	0.42	-
Undecan-2-one	1297	-	0.87	-	0.65	-	-	-	-	-	-
Undecanal	1311	-	0.62	-	0.27	-	-	-	-	-	-
(*E*)-Undec-2-en-1-ol	1347	-	0.98	-	1.21	-	-	-	-	-	-
α-Cubebene	1355	-	0.68	-	-	-	-	-	-	-	-
β-Bourbonene	1389	-	0.94	-	-	-	-	-	-	-	-
β-Cubebene	1393	-	0.93	-	-	-	-	-	-	-	-
Tetradecane	1402	-	0.91	-	0.61	-	0.89	-	-	-	-
6,10-Dimethylundecan-2-one	1408	-	0.49	-	0.42	-	-	-	-	-	-
α-Ionone	1432	-	0.82	-	0.69	0.74	-	0.64	-	0.23	-
Germacrene D	1485	5.44	-	-	-	0.85	-	-	-	-	-
β-Ionone	1490	6.22	2.50	3.84	3.01	3.62	1.34	5.38	1.28	1.97	-
Pentadec-1-ene	1495	4.68	2.22	13.82	2.36	25.14	1.93	23.70	4.53	53.17	9.54
(*E*)-Pentadec-7-ene	1498	0.70	-	1.00	0.45	1.29	-	1.96	-	3.56	4.88
Tridecan-2-one	1500	-	-	-	-	-	-	1.29	-	1.94	-
Pentadecane	1500	3.54	22.06	9.66	38.46	9.02	21.22	7.31	24.99	4.10	29.33
Tridecanal	1519	2.78	-	2.64	-	2.02	-	4.11	-	5.92	-
δ-Cadinene	1528	1.10	-	-	-	-	-	-	-	-	-
Dihydroactinidiolide	1533	-	1.26	-	1.27	-	-	-	-	-	-
Gleenol	1589	3.11	-	0.78	-	0.55	-	0.36	-	-	-
Hexadecane	1603	-	-	-	-	0.31	-	-	-	-	-
Heptadec-1-ene	1696	0.94	-	1.09	-	0.61	-	1.19	-	2.34	-
Heptadecane	1700	0.71	2.00	3.58	4.35	2.51	4.97	4.23	17.67	1.75	15.52
1-Phytene (3,7,11,15-Tetramethylhexadec-1-ene)	1791	-	-	-	-	2.49	1.82	-	-	-	-
Phytane	1813	-	-	-	-	3.95	4.46	-	-	-	-
Hexahydrofarnesyl acetone (Phytone)	1850	-	1.48	-	-	-	-	-	-	-	-

**Table 2:** The volatile compounds from *H. scoparia* isolated by headspace solid-phase microextraction (HS-SPME) and analysed by gas chromatography–mass spectrometry (GC–MS) using PDMS/DVB fibre (f2); Fr (fresh), Dr (dry).

Compound	RI	Area %									
		May		June		July		August		September	
		Fr	Dr	Fr	Dr	Fr	Dr	Fr	Dr	Fr	Dr
Dimethyl sulfide	<900	-	-	-	-	-	0.68	-	0.20	-	2.50
Butanal	<900	-	-	0.32	-	0.56	-	0.68	-	0.12	-
3-Methylbutanal	<900	0.51	-	0.77	-	0.61	-	0.70	-	0.16	-
Pent-1-en-3-ol	<900	1.69	1.31	1.35	2.70	0.86	1.74	1.14	1.03	0.72	3.97
Pentanal	<900	-	-	-	0.11	-	0.18	-	0.40	-	1.10
3-Hydroxybutan-2-one	<900	14.16	-	-	-	0.74	-	-	-	-	-
3-Methylbutanol	<900	1.18	-	0.20	-	0.64	-	0.57	-	-	-
2-Methylbutanol	<900	-	1.35	-	-	-	-	-	-	-	-
Pyridine	<900	-	-	-	-	-	-	0.15	-	0.19	-
(*E*)-Pent-2-enal	<900	-	-	-	-	-	0.44	-	-	-	-
Pentan-1-ol	<900	-	0.58	-	0.74	0.14	0.62	0.21	1.12	-	1.25
(*Z*)-Pent-2-en-1-ol	<900	0.42	0.40	0.30	0.80	0.24	0.79	0.32	0.61	0.19	1.82
Hexanal	<900	1.39	2.46	2.27	2.09	1.06	4.98	1.90	8.22	1.89	11.47
3-Methylbutanoic acid	<900	-	1.94	-	2.43	-	-	-	-	-	-
2-Methylbutanoic acid	<900	-	1.58	-	1.58	-	-	-	-	-	-
(*E*)-Hex-2-enal	<900	-	0.93	0.59	1.12	0.19	1.97	0.37	1.48	0.23	-
Hexan-1-ol	<900	4.60	2.47	-	1.08	1.79	0.97	1.58	1.78	-	-
Heptan-2-one	<900	-	0.31	-	0.30	-	0.28	-	-	-	-
Tribromomethane	<900	0.25	-	0.58	-	0.49	-	0.18	-	0.46	-
Heptanal	907	-	1.94	-	1.31	-	3.94	-	10.04	-	8.14
6-Methylheptan-2-one	962	5.85	0.52	0.71	0.43	1.00	0.58	1.08	-	-	-
Benzaldehyde	970	9.51	5.11	52.75	3.82	32.99	4.82	57.85	2.62	28.39	6.83
Heptan-1-ol	975	-	0.80	-	0.97	-	0.71	-	0.72	-	-
Oct-1-en-3-one	979	0.87	-	0.80	-	0.60	-	0.37	-	0.39	-
Pentyl propanoate	980	-	-	-	4.12	-	1.61	-	-	-	-
Hexanoic acid	982	-	4.80	-	-	-	-	-	-	-	-
Oct-1-en-3-ol	984	1.81	5.82	1.01	8.56	0.71	5.30	0.68	2.19	0.51	2.74
Octan-3-one	991	1.73	-	0.82	-	0.39	-	0.30	-	-	-
6-Methylhept-5-en-2-one	992	-	1.30	-	1.18	-	0.80	-	-	-	-
2-Pentylfuran	995	-	0.85	0.33	1.01	0.31	1.73	0.59	1.34	0.35	-
Octanal	1007	0.88	1.26	0.66	0.93	-	3.22	-	4.28	-	-
(*E,E*)-Hepta-2,4-dienal	1016	-	-	-	0.47	-	1.06	-	-	-	-
2-Ethylhexan-1-ol	1035	-	-	-	0.55	-	2.16	-	2.27	-	-
(*E*)-3-Ethyl-2-methylhexa-1,3-diene	1038	-	1.62	0.69	1.60	1.51	1.39	-	-	0.15	-
Benzyl alcohol	1041	3.67	6.53	0.87	1.61	0.61	5.18	2.86	2.05	-	-
(*E*)-β-Ocimene	1044	-	3.55	-	1.42	-	2.57	-	1.99	-	-
Phenylacetaldehyde	1052	0.99	0.73	-	-	-	1.67	-	-	-	-
γ-Caprolactone	1062	-	1.17	-	0.48	-	-	-	-	-	-
(*E*)-Oct-2-enal	1064	-	-	-	0.65	-	1.74	-	0.90	-	-
(*E*)-Oct-2-en-1-ol	1074	-	0.50	-	0.87	-	0.79	-	-	-	-
Octan-1-ol	1076	13.93	-	3.00	-	0.80	-	-	-	-	-
(*E,E*)-Octa-3,5-dien-2-one	1077	-	0.95	-	1.35	-	2.75	-	1.84	-	-
Nonan-2-one	1096	0.53	-	-	-	-	-	-	-	-	-
(*E,Z*)-Octa-3,5-dien-2-one	1097	-	0.99	-	1.55	-	1.32	-	0.40	-	-
Nonanal	1108	-	2.52	0.53	0.65	-	4.97	-	4.29	-	5.10
2,6-Dimethylcyclohexan-1-ol	1114	1.75	5.72	1.35	5.34	0.96	2.05	0.62	3.25	0.76	-
4-Ketoisophorone	1150	-	2.27	0.54	1.13	1.01	0.41	-	-	0.61	-
(*Z*)-Non-3-en-1-ol	1155	3.76	-	-	-	-	-	-	-	-	-
6-[(1*Z*)-Butenyl]-cyclohepta-1,4-diene] (Dictyopterene D’)	1158	1.23	0.35	0.37	0.33	0.59	-	0.57	-	-	-
[6-Butyl-cyclohepta-1,4-diene] (Dictyopterene C’)	1175	-	-	-	-	-	-	0.31	-	-	-
Decan-2-one	1196	-	-	-	-	-	-	0.38	-	-	-
Decanal	1209	-	0.33	-	0.21	-	0.90	-	-	-	-
β-Cyclocitral	1226	1.81	0.63	1.09	0.61	0.81	0.70	0.81	-	0.60	-
Undecan-2-one	1296	1.05	0.47	-	0.37	-	0.61	-	-	-	-
Undecanal	1311	-	0.66	-	0.24	-	-	-	-	-	-
(*E*)-Undec-2-en-1-ol	1347	-	0.68	-	0.97	-	-	-	-	-	-
α-Cubebene	1355	-	0.53	-	-	-	-	-	-	-	-
β-Bourbonene	1389	-	0.62	-	-	-	-	-	-	-	-
β-Cubebene	1393	-	0.48	-	-	-	-	-	-	-	-
Tetradecane	1400	-	0.57	-	0.48	-	0.67	-	-	-	-
6,10-Dimethylundecan-2-one	1409	-	0.35	-	0.40	-	-	-	-	-	-
Dodecanal	1412	1.67	-	-	-	-	-	-	-	-	-
α-Ionone	1432	-	0.30	-	0.56	0.53	-	0.41	-	0.43	-
Germacrene D	1485	1.87	-	0.25	-	0.95	-	-	-	-	-
β-Ionone	1489	3.67	1.96	3.57	2.43	2.87	1.39	2.96	1.42	2.84	-
Pentadec-1-ene	1495	2.15	1.59	7.88	2.04	23.77	1.92	10.36	4.00	40.35	6.90
(*E*)-Pentadec-7-ene	1498	0.47	-	0.64	0.41	1.16	-	0.97	-	3.63	-
Tridecan-2-one	1500	-	-	-	-	0.44	-	0.50	-	1.63	-
Pentadecane	1500	1.95	14.06	4.91	27.13	6.41	17.01	2.41	20.64	2.68	24.47
Tridecanal	1514	3.29	-	4.34	-	4.44	-	4.89	-	8.00	-
Dihydroactinidiolide	1533	0.18	1.16	0.31	1.04	0.22	-	0.16	-	-	-
Gleenol	1589	2.23	-	0.51	-	0.33	-	-	-	-	-
Hexadecane	1600	-	-	-	-	-	0.88	-	-	-	-
Heptadec-1-ene	1696	-	-	0.53	-	0.57	-	0.38	-	1.64	-
Heptadecane	1703	0.56	1.69	1.48	3.76	2.06	4.95	0.84	18.29	1.05	23.72
Phytane	1813	-	-	-	-	-	4.19	-	-	-	-
Hexahydrofarnesyl acetone (Phytone)	1850	-	1.22	-	-	-	0.67	-	-	-	-

**Table 3:** The volatile compounds from *H. scoparia* acquired by hydrodistillation (HD) and analysed by gas chromatography–mass spectrometry (GC–MS); Fr (fresh), Dr (dry).

Compound	RI	Area %									
		May		June		July		August		September	
		Fr	Dr	Fr	Dr	Fr	Dr	Fr	Dr	Fr	Dr
2-Ethyl-5,5-dimethylcyclopenta-1,3-diene	<900	0.06	0.14	0.10	0.23	0.02	0.09	0.02	0.22	0.10	0.19
(*E*)-Hex-2-enal	<900	1.71	1.18	1.13	1.00	0.13	0.31	0.27	0.78	1.76	1.18
Hex-3-en-1-ol	<900	0.06	-	-	-	0.14	-	-	-	-	-
Hexan-1-ol	<900	1.72	0.17	0.81	0.18	0.18	-	0.22	0.04	0.24	0.03
*m*-Xylene	<900	0.51	-	-	-	0.06	-	-	-	0.13	-
2,3-Dimethylpyridine	<900	0.04	-	-	-	-	-	-	-	-	-
Heptan-2-one	<900	0.16	0.14	0.15	0.30	-	0.12	-	0.24	-	0.28
Tribromomethane	<900	-	-	-	-	0.09	-	0.03	-	0.34	-
Hept-4-enal	902	0.24	0.14	0.08	-	-	-	-	-	0.16	0.10
Heptanal	904	0.06	0.71	0.10	1.11	0.06	0.25	-	0.34	0.07	0.21
(*E,Z*)-Hexa-2,4-dienal (Sorbaldehyde)	914	0.06	-	-	-	-	-	0.04	-	-	-
2-Iodopentane	929	0.12	-	0.09	-	-	-	-	-	0.04	-
2,4-Dimethylpyridine	937	0.16	-	0.09	-	0.03	-	-	-	-	-
6-Methylheptan-2-one	960	0.05	-	0.11	-	-	-	-	-	-	-
(*E*)-Hept-2-enal	962	-	0.06	-	0.17	-	-	-	0.14	0.09	0.07
Benzaldehyde	969	0.73	0.69	0.75	0.95	0.17	0.45	0.13	0.41	0.30	0.47
Heptan-1-ol	973	0.13	-	0.09	-	-	-	-	-	-	-
Dimethyl trisulfide	978	0.22	0.22	0.36	0.33	0.39	0.11	0.05	0.11	0.13	0.08
Oct-1-en-3-ol	982	0.34	0.35	0.48	0.73	0.09	0.40	0.04	0.23	0.24	0.28
Octan-2,3-dione	987	0.18	0.34	0.53	0.76	0.15	0.48	0.25	0.57	0.90	0.16
Octan-3-one	989	0.38	0.24	0.67	0.44	0.08	-	-	0.16	0.21	-
Octan-2-one	994	-	-	0.16	-	-	-	-	-	-	-
2-Pentylfuran	995	0.43	0.88	0.17	1.16	0.09	0.38	0.11	0.69	0.90	0.77
Octan-3-ol	998	-	-	0.23	0.22	-	-	-	0.15	-	0.10
(*E*,*Z)*-Hepta-2,4-dienal	1000	-	-	-	-	0.06	-	0.08	-	0.11	-
Octanal	1005	0.25	0.33	0.19	0.33	-	0.17	-	0.11	0.08	0.12
(*E,E*)-Hepta-2,4-dienal	1015	0.24	0.30	0.30	0.50	0.14	0.17	0.21	0.12	0.23	0.14
*p*-Cymene	1030	0.06	-	-	-	-	-	-	-	-	-
2-Ethylhexan-1-ol	1033	-	-	-	-	-	0.07	-	0.23	0.04	-
(3*E*)-3-Ethyl-2-methylhexa-1,3-diene	1039	-	-	-	-	-	-	-	-	0.05	-
Benzyl alcohol	1041	0.18	-	0.19	-	0.06	-	-	-	0.04	-
2,2,6-Trimethylcyclohexan-1-one	1042	-	0.13	-	0.22	-	-	-	-	-	-
Phenylacetaldehyde	1051	0.15	0.77	0.32	0.99	0.20	0.57	0.17	0.33	0.25	0.27
(*E*)-Oct-2-enal	1063	0.14	0.26	0.13	0.52	0.05	0.21	-	0.32	0.32	0.35
2-Methyldecane	1068	0.11	-	0.08	-	0.06	-	0.12	-	0.50	-
(*E*)-Oct-2-en-1-ol	1073	0.20	-	0.15	-	0.08	-	0.05	-	0.13	-
Acetophenone	1073	-	0.21	-	0.25	-	0.16	-	0.17	-	0.20
Octan-1-ol	1075	3.82	-	1.79	-	0.25	-	0.10	-	0.39	-
(*E,E*)-Octa-3,5-dien-2-one	1075	-	0.31	-	0.58	-	0.20	-	0.20	-	0.25
1-Methylsulfanylpentan-3-one	1091	-	0.31	-	0.29	-	0.22	-	0.15	-	0.08
(*E,Z*)-Octa-3,5-dien-2-one	1096	1.95	0.17	0.91	0.25	0.49	-	0.55	-	1.16	0.18
Linalool	1103	-	-	-	0.11	-	-	-	-	0.13	0.09
Nonanal	1107	0.11	0.44	0.12	0.61	-	0.28	-	0.24	0.18	0.21
2,6-Dimethylcyclohexan-1-ol	1113	0.15	0.34	0.11	0.54	0.08	0.37	0.10	0.21	0.29	0.24
2-Phenylethanol	1120	0.10	-	0.10	-	0.73	-	-	-	0.08	-
(*E,Z*)-2,6-Dimethylocta-1,3,5,7-tetraene	1141	0.21	-	0.08	-	-	-	-	-	-	-
4-Ketoisophorone	1150	0.56	0.56	0.34	0.24	0.17	0.39	0.09	0.31	0.13	0.26
(*E,Z*)-Nona-2,6-dienal	1159	0.30	0.17	0.22	0.32	0.17	0.08	-	0.15	0.22	0.16
6-[(1*Z*)-Butenyl]-1,4-cycloheptadiene] (Dictyopterene D’)	1161	-	-	-	-	-	-	-	-	0.06	-
Non-2-enal	1165	0.14	0.16	0.11	0.33	0.18	0.09	-	0.14	0.20	0.19
[6-Butyl-1,4-cycloheptadiene] (Dictyopterene C’)	1175	0.14	-	-	-	0.10	-	-	-	-	-
2,4-Dimethylbenzaldehyde	1180	0.10	0.31	0.17	0.53	-	-	0.05	0.13	0.15	0.11
3,4,4-Trimethylcyclohex-2-en-1-one	1194	-	-	-	-	-	-	-	0.19	-	0.19
Dodecane	1200	-	0.12	-	0.25	-	-	-	-	-	-
Decanal	1209	0.38	-	0.21	0.15	-	0.11	-	0.09	0.07	0.09
β-Cyclocitral	1225	0.28	0.36	0.15	0.53	-	0.11	-	0.21	0.24	0.18
β-Cyclohomocitral	1263	0.17	0.21	0.14	0.34	-	-	-	0.11	0.18	0.10
(*E,Z*)-Deca-2,4-dienal	1283	0.15	0.16	-	0.23	0.05	0.18	0.08	0.10	0.24	0.10
Indole	1296	0.19	0.36	0.17	0.27	0.13	0.25	0.08	0.26	0.35	0.22
Undecanal	1310	-	0.13	-	0.25	0.06	0.18	-	0.22	0.20	0.28
(*E,E*)-Deca-2,4-dienal	1320	0.38	0.80	0.48	1.98	0.15	0.40	0.25	0.75	1.03	0.54
(*E*)-Undec-2-en-1-ol	1347	0.11	0.27	-	0.30	-	0.18	-	0.15	0.07	0.08
β-Bourbonene	1388	0.19	-	0.09	-	-	-	-	-	-	-
β-Cubebene	1393	0.42	0.13	0.46	0.40	0.15	-	-	-	0.16	0.21
4-(2-Methylbutan-2-yl)phenol (*p*-*tert-*Pentylphenol)	1400	-	-	-	-	0.04	-	-	-	0.07	-
Tetradecane	1400	0.27	0.50	0.26	0.66	0.07	0.23	0.09	1.18	0.91	0.89
6,10-Dimethylundecan-2-one	1408	-	0.20	-	0.21	-	-	-	0.09	-	0.12
Dodecanal	1412	-	0.21	-	0.38	0.04	0.19	-	0.22	0.18	0.38
α-Ionone	1432	0.13	0.26	0.12	0.41	0.05	0.14	0.07	0.27	0.30	0.31
(*E*,*Z*)-Dodeca-2,6-dienal	1452	-	-	-	-	-	-	-	0.04	-	0.10
(*Z*)-Geranylacetone	1458	0.30	0.35	-	0.44	-	-	-	0.13	0.14	0.18
Dodecan-1-ol	1479	1.38	-	0.13	-	-	0.71	-	0.12	0.11	0.35
α-Muurolene	1481	-	-	-	0.37	-	-	-	-	-	0.10
Germacrene D	1485	2.67	0.24	1.71	1.67	0.56	-	0.06	0.34	0.39	0.59
β-Ionone	1489	2.13	5.04	0.86	5.56	0.56	2.14	0.70	2.94	2.96	3.72
Pentadec-1-ene	1495	7.70	2.99	7.56	8.53	4.41	1.68	2.88	17.50	26.46	18.98
(*E*)-Pentadec-7-ene	1499	0.80	0.58	0.66	1.33	0.37	0.35	0.30	2.00	3.26	3.10
Tridecan-2-one	1500	0.18	0.15	-	0.34	0.11	0.08	0.14	0.83	1.41	1.23
Pentadecane	1500	4.26	1.19	4.54	3.80	1.63	0.61	0.74	1.66	2.94	1.47
Tridecanal	1514	0.80	0.99	1.11	1.92	0.79	0.80	0.54	1.88	3.11	2.74
Zonarene	1530	0.42	0.13	0.36	0.46	0.16	0.17	-	0.03	-	0.26
Dihydroactinidiolide	1532	-	0.17	-	-	-	-	-	-	-	-
Tetradecan-2-one	1565	-	0.15	-	-	-	-	-	-	-	-
Dodecanoic acid	1573	-	0.19	0.19	0.27	-	0.18	0.10	0.17	0.19	0.21
Tridecan-1-ol	1580	0.28	0.40	0.04	0.10	0.18	0.22	0.08	0.11	-	0.25
Gleenol	1589	6.26	0.39	3.91	0.81	1.57	0.10	0.33	0.26	0.82	1.30
Hexadec-1-ene (Cetene)	1595	-	-	-	-	-	-	-	-	-	0.11
Diethyl phthalate	1597	-	-	-	-	-	-	0.06	0.05	0.10	0.24
Hexadecane	1600	-	-	-	-	-	0.46	-	-	-	0.30
Tetradecanal	1616	0.25	0.42	0.33	0.73	0.14	0.38	0.14	0.32	0.30	0.48
(*Z*)-Hexadec-7-ene	1622	-	-	0.06	0.14	-	-	0.08	0.35	0.23	0.33
Benzophenone	1630	-	-	-	-	0.11	0.42	0.10	0.14	-	-
Cubenol	1647	0.18	-	0.09	-	-	-	-	-	-	-
(*E*)-Heptadec-8-ene	1678	-	0.18	0.24	0.36	-	0.43	0.21	0.89	0.49	0.80
Tetradecan-1-ol	1681	0.59	1.70	0.74	1.87	0.25	1.54	0.18	0.85	0.39	0.94
(*Z*)-Pentadec-6-en-1-ol	1690	3.92	1.40	1.49	2.03	0.60	0.33	0.22	0.84	0.69	0.87
Heptadec-1-ene	1696	2.27	0.68	2.06	1.60	1.57	0.59	1.66	2.91	6.82	5.67
Heptadecane	1700	1.97	1.12	2.74	2.44	2.75	0.93	1.05	2.15	2.58	1.70
2,6,10,14-Tetramethyl-7-(3-methylpentyl)pentadecane	1709	0.18	-	-	-	0.26	-	-	-	-	-
Pentadecanal	1718	1.21	2.03	3.00	2.95	2.53	1.61	2.46	2.12	2.35	1.68
(*Z*)-Heptadec-3-ene	1720	-	-	-	-	-	-	-	-	0.76	0.57
Tetradecanoic acid	1772	-	1.05	-	-	-	-	-	-	-	-
Pentadecan-1-ol	1782	0.29	-	0.20	-	0.26	-	-	-	-	0.20
Octadec-1-ene	1786	-	1.03	-	1.24	-	0.72	-	0.44	-	0.18
Octadecane	1800	-	-	-	-	0.14	-	-	-	-	-
2-Phenoxyethoxybenzene	1807	-	-	0.08	-	-	-	0.07	-	-	0.08
Phytane	1813	-	-	-	-	6.11	2.43	-	-	-	-
Hexadecanal	1820	-	0.17	0.24	0.35	0.15	0.53	0.09	0.15	-	0.12
6,10,14-Trimethylpentadecan-2-one	1850	1.19	6.77	2.27	5.48	3.84	7.58	1.69	5.08	2.68	5.27
*p*-Cumylphenol	1855	0.34	0.85	0.51	0.61	0.29	0.84	0.23	0.89	0.24	0.62
(*Z*)-Hexadeca-1,9-diene	1864	0.23	1.58	-	1.48	0.18	0.87	0.08	0.66	-	0.25
(*Z*)-Hexadec-11-enal	1868	-	-	0.33	-	0.82	-	0.06	-	-	-
Diisobutyl phthalate	1872	-	0.85	0.21	1.04	0.43	2.40	0.20	0.71	-	0.67
Hexadecan-1-ol	1888	-	2.97	0.73	4.22	-	4.76	0.20	1.97	-	1.04
Nonadec-1-ene	1896	0.67	0.93	2.33	1.38	3.42	1.10	1.21	1.17	0.56	0.80
Nonadecane	1900	0.29	-	-	-	0.52	-	0.15	-	-	-
Heptadecan-2-one	1905	-	0.13	0.20	0.32	-	0.28	0.22	0.24	-	0.31
(*E,E*)-Farnesyl acetone	1923	-	0.98	0.40	0.87	0.46	1.07	0.24	0.80	0.25	0.36
Isophytol	1953	-	0.41	0.17	0.27	0.25	0.37	0.11	0.23	-	0.10
Palmitoleic acid	1963	-	0.23	-	0.19	-	0.09	-	-	-	-
Dibutyl phthalate	1967	-	0.26	-	0.74	-	4.29	-	0.29	-	0.37
Hexadecanoic acid	1973	0.19	4.80	0.93	0.20	0.25	0.08	0.45	0.47	0.21	0.61
(*Z*)-Octadecen-9-al	1999	0.76	1.84	1.68	1.29	2.54	1.58	8.51	3.45	3.05	3.52
Octadecanal	2024	0.14	1.26	0.48	1.66	0.63	2.44	0.43	1.39	-	0.39
(*Z*)-Falcarinol	2032	0.26	-	-	-	0.55	0.54	0.73	0.70	0.35	0.85
Methyl (all *Z*) eicosa-5,8,11,14,17-pentaenoate	2038	0.15	0.98	0.48	0.41	0.22	0.38	0.20	0.27	-	0.24
Methyl (all *Z*) eicosa-5,8,11,14-tetraenoate	2044	2.85	2.86	1.86	1.36	1.51	0.91	0.83	0.68	0.46	0.99
(*Z,Z*)-Octadeca-9,12-dien-1-ol	2050	1.90	0.66	2.03	0.62	2.51	0.97	1.87	0.55	0.44	0.15
(*Z,Z,Z*)-9,12,15-Octadecatrien-1-ol	2056	8.09	0.86	4.63	0.66	6.67	0.56	2.84	0.45	0.47	0.10
(*Z*)-Octadec-9-en-1-ol	2061	0.18	0.69	0.25	0.23	0.54	1.01	0.35	0.42	-	0.25
(*Z,Z*)-Octadeca-3,13-dien-1-ol	2065	-	0.87	-	0.28	-	1.67	-	0.68	-	-
Heneicos-10-ene	2070	-	1.32	-	0.49	-	0.11	-	-	-	-
Octadecan-1-ol	2088	-	-	0.13	-	0.41	-	0.53	-	-	-
γ-Palmitolactone	2104	-	1.22	0.55	0.39	0.97	1.75	1.73	0.55	-	0.37
(*Z,Z*)-Octadeca-9,12-dienoic acid	2110	-	-	0.42	-	1.42	-	1.26	-	0.16	-
*(E)*-Phytol	2116	11.35	9.31	18.71	8.22	23.25	26.10	47.03	22.53	8.01	12.87
Isopachydictyol A	2127	2.73	9.70	3.07	2.60	2.80	2.25	1.57	1.22	0.96	3.54
(*Z*)-Octadec-9-enoic acid (Oleic acid)	2142	0.65	1.85	1.60	0.56	1.21	1.51	2.01	1.20	0.47	1.32
Cembra-4,7,11,15-tetraen-3-ol	2230	3.06	6.88	3.63	3.00	3.94	4.18	1.67	1.30	0.86	4.11

## Data Availability

Data are contained within the article.

## Sample Availability

Samples of the compounds are not available from the authors.
